# Worse prognosis in females with new onset of depression after oral cancer diagnosis: a retrospective case-control study

**DOI:** 10.3389/fonc.2023.1248926

**Published:** 2023-10-12

**Authors:** Elena Hofmann, Christian Doll, Alize Rogge, Robert Preissner, Max Heiland, Saskia Preissner, Steffen Koerdt

**Affiliations:** ^1^ Department of Oral and Maxillofacial Surgery, Charité – Universitätsmedizin Berlin, Corporate Member of Freie Universität Berlin and Humboldt-Universität zu Berlin, Berlin, Germany; ^2^ Berlin Institute of Health (BIH) Charité Junior Clinician Scientist Program, Berlin Institute of Health at Charité – Universitätsmedizin Berlin, Berlin Institute of Health Biomedical Innovation Academy, Berlin, Germany; ^3^ Centre for Patient-Centered Outcomes Research, Charité – Universitätsmedizin Berlin, Corporate Member of Freie Universität, Berlin, Humboldt-Universität zu Berlin and Berlin Institute of Health, Berlin, Germany; ^4^ Institute of Physiology and Science-IT, Charité - Universitätsmedizin Berlin, Corporate Member of Freie Universität Berlin, Humboldt-Universität zu Berlin and Berlin Institute of Health, Berlin, Germany

**Keywords:** gender aspects, sex, female, oral cancer, real-world data, prognostic factors, depression

## Abstract

**Background:**

Sex-related discrepancies in the prognosis of oral cancer patients have not been clarified. This study aimed to assess survival outcomes and potential prognostic factors in female and male patients with oral cancer.

**Methods:**

A retrospective search of the TriNetX network (TriNetX, Cambridge, Massachusetts, USA) was conducted to identify patients diagnosed with oral cancer (International Classification of Diseases (ICD)-10 codes C02–C06), within the past 20 years from the access date April 21, 2023. Patients were categorized according to sex (female vs. male). Following matching for age and risk factors such as nicotine dependence and alcohol abuse, Kaplan-Meier analysis was performed and risk, odds, and hazard ratios were calculated. Outcome variables were five-year disease-free survival (DFS) and overall survival (OS). Additionally, the female and male patient cohort were compared with regard to the novel diagnosis of depression (depressive episode, major depressive disorder, dysthymic disorder) after the tumor diagnosis.

**Results:**

A total of 77,348 patients were assessed. After propensity score matching, 26,578 male and 26,578 female patients were included in each group (mean age 63 years). DFS (71.92% in females vs. 68.29% in males; hazard ratio (HR) 0.870; *p* < 0.001) and OS (77.08% in females vs. 71.74% in males; HR 0.793; *p* < 0.001) were significantly higher in the female cohort. However, in patients diagnosed with depression after the initial cancer diagnosis (N = 4,824), survival was worse in female patients compared to male patients (82.48% in females vs. 86.10% in males; HR 1.341; *p* < 0.001).

**Conclusion:**

This retrospective case-control study showed that females with oral cancer had a better DFS and OS than males. However, survival in females with a newly diagnosed depression after the oral cancer diagnosis was worse compared to those of male oral cancer patients. Depression may be a relevant prognostic factor that contributes to sex disparities in oral cancer patients.

## 1 Introduction

Overall incidence rates for cancers worldwide were 19% higher in males than in females in 2020, with an even greater difference of 43% in overall cancer mortality between male and female patients (1). The discrepancy in incidence and death rates highlights the need to consider sex-related aspects in cancer patients, including those affected by oral cancer (1).

Based on a national database query in the United States, worse survival was reported in male patients affected by cancers of the lip, larynx, hypopharynx, esophagus and urinary bladder (2). Overall survival (OS) was better in women than in men affected by laryngeal squamous cell carcinoma (3) and human papilloma virus (HPV)-positive oropharyngeal squamous cell carcinoma (OPSCC) (4). In the field of head and neck cancers, HPV is a well-studied driver for the development of OPSCC (5) and HPV-status is intimately linked to sexual behavior (6). In HPV-negative OPSCC, Hunter et al. reported worse outcomes in female patients with regard to disease-specific survival (DSS) and OS, as compared to male patients (7). A recent study demonstrated worse OS and DFS in females with early-stage tongue squamous cell carcinoma (8). In contrast, female sex was identified as a significant positive prognostic factor in oral squamous cell carcinoma (OSCC) in a retrospective study, but the five-year disease-free survival (DFS) showed no statistically significant difference between the female and male cohort (9). Reasons for gender-related discrepancies in survival outcomes remain a source of discussion. Possible relevant factors may be differences in sex hormones and genetic profile as well as differences in risk behavior between female and male patients (10), but there may be other causes.

A high proportion of cancer patients, especially young females, present with clinical symptoms of depression, emotional distress and anxiety (11, 12). However, the prognostic role of depression in cancer patients, including oral cancer patients, has not been determined (13). Recent studies suggested that mental health disorders such as depression and anxiety may be associated with worse cancer survival due to delayed treatment and worse treatment adherence (14). Poor compliance and non-adherence to recommended therapies in patients with mental health disorders may be important factors for long-term survival outcomes and may serve as a potential explanation for the link between depression and cancer prognosis. Differences in survival outcomes between female and male oral cancer patients with depression in a large patient cohort have not been clarified.

Research efforts in various tumor entities have previously been directed at sex-related aspects in cancers, given that risk factors, lifestyle choices, genetic predisposition and hormones vary between women and men (10). Data on discrepancies in oral cancer patients are controversial and contributing factors have not been clarified. A retrospective case-control study was conducted based on data retrieved from the TriNetX platform to compare survival outcomes between female and male oral cancer patients. Up-to-date real-world data on more than 250 million patients from more than 120 health care organizations (HCOs) worldwide can be accessed via the TriNetX global health research network (TriNetX, Cambridge, Massachusetts, USA). The objective of this study was to assess the role of sex as a prognostic factor in oral cancer patients in a large patient cohort and to evaluate the potential influences of depression.

## 2 Patients and methods

### 2.1 Data acquisition, inclusion and exclusion criteria

The TriNetX network was accessed on April 21, 2023. This query was run on the COVID-19 Research Network, the platform of a group of 80 HCOs. The database was searched for the electronic medical records of patients at least 18 years of age, diagnosed with oral cancer according to the International Classification of Diseases (ICD)-10 codes C02–C06, thus including malignant neoplasms of the tongue, gum, floor of mouth, palate and other parts of the mouth, up to 20 years before the access date (2003–2023). Patient records were searched for female and male sex and risk factors such as nicotine dependence (Z87.891) and alcohol abuse (F10.1). Other factors were retrieved according to ICD-10 codes, such as secondary malignant neoplasms (C79), secondary lymph node metastasis (C77), secondary malignant neoplasm of respiratory and digestive organs (C78), depressive episode (F32), major depressive disorder (F33) and dysthymic disorder (F34.1).

**Figure 1:**
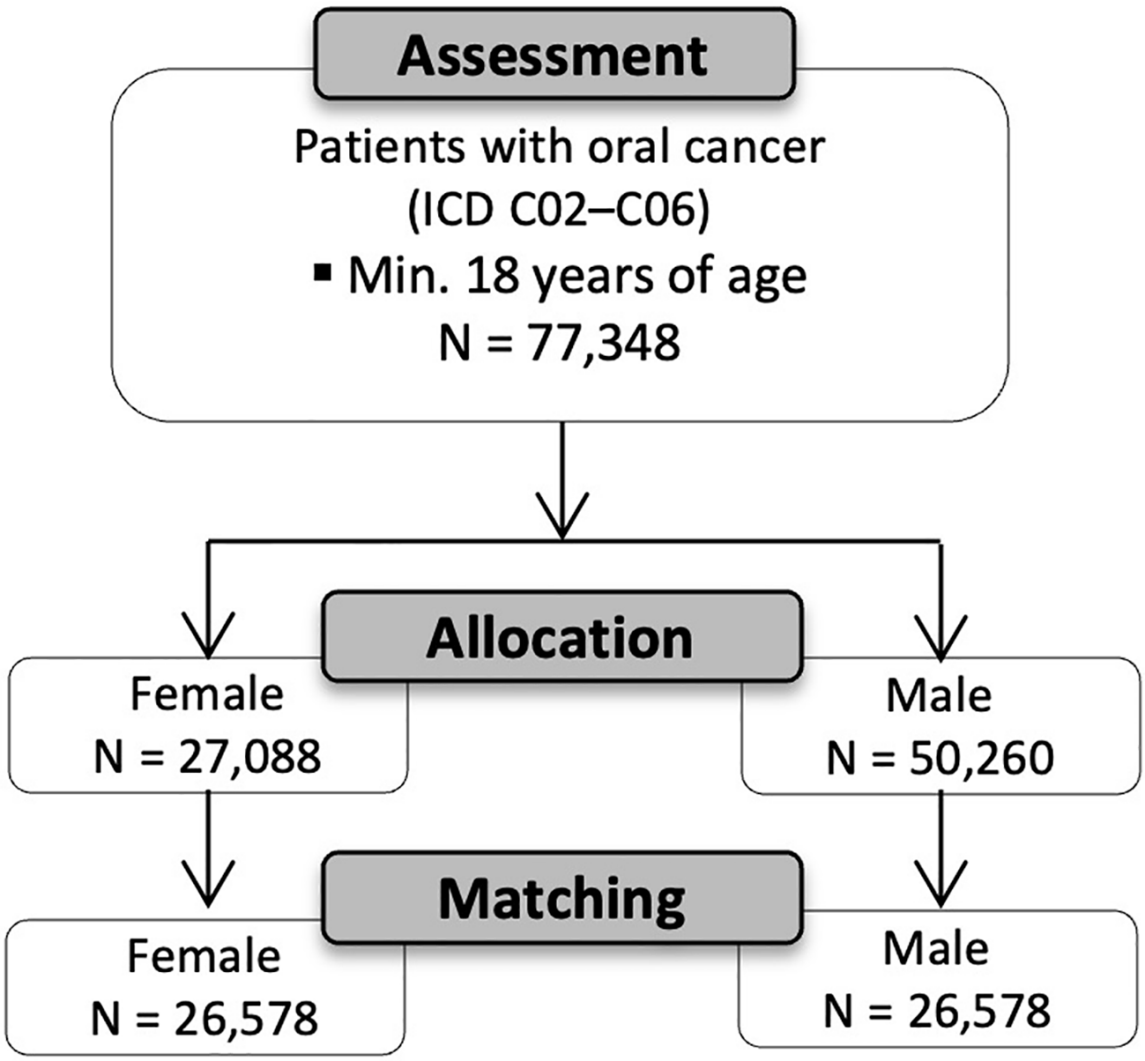
Modified CONSORT flow chart (15).

**Figure 1** displays the modified Consolidated Standards of Reporting Trials (CONSORT) flow chart (15). Patients were grouped according to sex (male vs. female). The patient count in the female cohort was 27,088 patients from 73 HCOs before matching and the male cohort included 50,260 males from 72 HCOs. Propensity score matching was applied to reduce confounding variables and equate groups based on similar covariate distributions. One-to-one matching was performed according to characteristics such as age at initial diagnosis and risk factors, including smoking and alcohol abuse. Following propensity score matching for age, smoking and alcohol abuse, 26,578 patients were included in the female and male cohort, respectively.

### 2.2 Data analysis

The point of entry for analysis was defined as the day of initial diagnosis. The observation period was five years following the initial diagnosis, since this time period was considered as the most relevant timeframe in terms of treatment outcomes. The outcome variables included disease-free survival (DFS) and overall survival (OS). In this analysis, DFS was defined as the time period from the day of the initial cancer diagnosis until the occurrence of a secondary malignant neoplasm (C79), secondary lymph node metastasis (C77), or secondary malignant neoplasm of respiratory and digestive organs (C78), as defined by ICD-10 codes. OS was defined from the day of initial diagnosis until the reported date of death. Furthermore, the groups were compared according to the new diagnosis of depression (i.e., depressive episode (F32), major depressive disorder (F33) and dysthymic disorder (F34.1)) after initial tumor diagnosis. Patients that had the outcome (i.e., secondary malignant neoplasm, death, depression) before the start of the time window, which was defined as the day of the initial cancer diagnosis, were excluded from the risk and survival analyses. These excluded cases were attributed to patients, in which the cancer diagnosis was recorded after the defined outcome variable (i.e., death) had occurred at an HCO included in the TriNetX database. Propensity score matching was conducted. Statistical analysis included a risk analysis and Kaplan-Meier survival analysis. Risk difference, risk ratio (RR), odds ratio (OR), hazard ratio (HR) and log-rank test were calculated with a 95% confidence interval (CI) to compare treatment outcomes between two groups. *P*-values below 0.05 were defined as statistically significant. Graphs were created using GraphPad Prism version 9 (La Jolla, CA, USA).

## 3 Results

### 3.1 Assessment, allocation, and matching

A total of 77,348 patients who were diagnosed with oral cancer according to ICD-10 codes C02–06 were assessed. Patients were grouped according to sex (male vs. female). The female cohort included 27,088 patients with a mean age of 63.2 years ± 15.7 SD from 73 HCOs. The male cohort included 50,260 patients from 72 HCOs. The mean age was 61.8 years ± 12.9 SD. The difference in age was statistically significant (*p* < 0.001). The analyses of the risk factors between the female and male cohort revealed a statistically significant difference in nicotine dependence between females (7.1%) and males (9.6%; *p* < 0.001). Furthermore, alcohol abuse was more common in the male cohort (4.1%) than in the female cohort (2.1%; *p* < 0.001). After propensity score matching, each cohort included 26,578 patients with a mean age of 62.7 years in the female cohort and 63.0 years in the male cohort. **Table 1** shows the patient characteristics of both cohorts before and after matching.

**Table 1:** Patient characteristics of the female (N=27,088) and the male (N=50,260) cohort before and after propensity score matching.

	Before matching				After matching			
	Female	Male	*P*-value	Std. mean difference	Female	Male	*P*-value	Std. mean difference
Patients (N)								
Total	27,088	50,260			26,578	26,578		
Nicotine dependence	1,935 (7.1%)	4,806 (9.6%)	<0.001	0.088	1,928 (7.3%)	1,913 (7.2%)	0.802	0.002
Alcohol abuse	566 (2.1%)	2,076 (4.1%)	<0.001	0.118	560 (2.1%)	660 (2.5%)	0.004	0.025
Age at index								
Mean (years)	63.2	61.8	<0.001	0.096	62.7	63.0	0.027	0.019
SD	15.7	12.9			15.4	15.1		

Percentage refers to the respective cohorts. P-value refers to the comparison between both cohorts (log-rank test). Std, Standardized; SD, Standard deviation.

### 3.2 Risk analysis and patient survival

**Figure 2:**
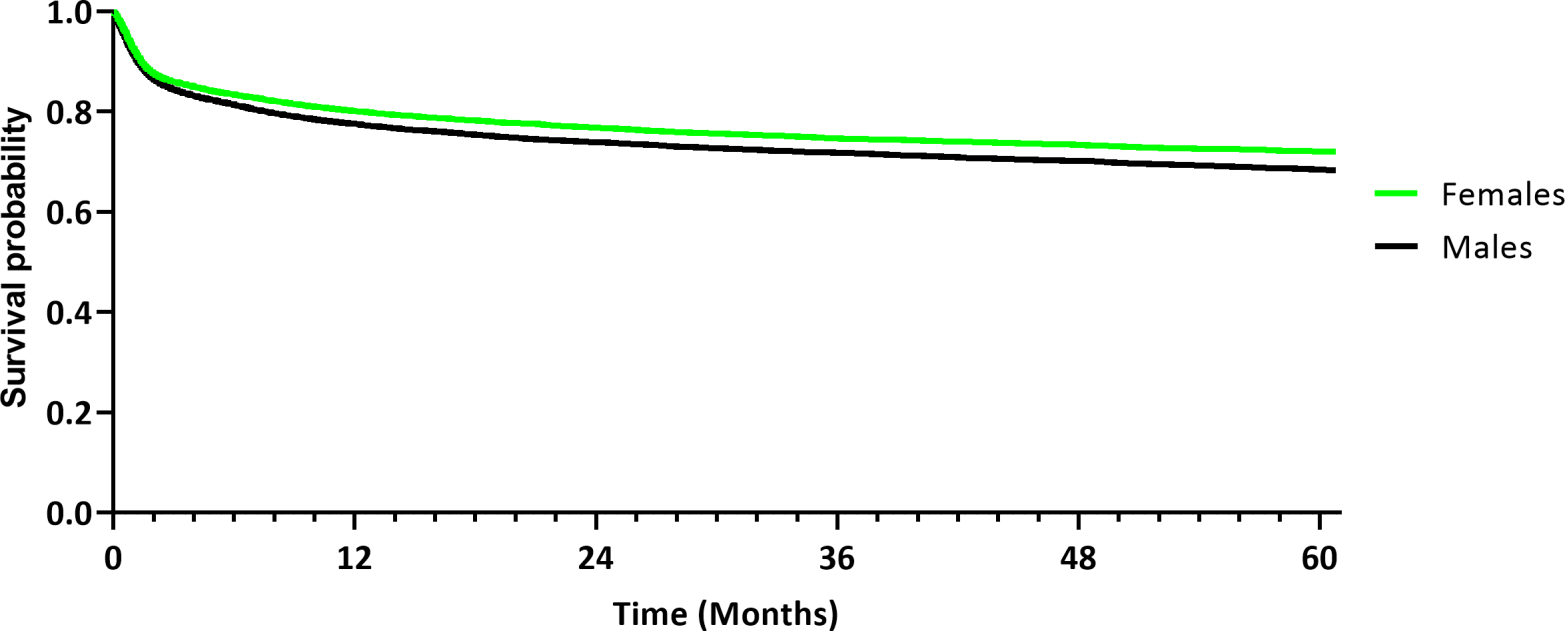
Kaplan-Meier curve demonstrating disease-free survival of the female and male cohort after propensity score matching. The outcome was defined as the occurrence of secondary malignancy or lymph node metastasis.

Statistical analysis was performed to compare five-year DFS between females and males, as demonstrated in **Table 2**. The occurrence of secondary malignant neoplasms, secondary lymph node metastasis or secondary malignant neoplasm of respiratory and digestive organs was reported in 5,121 female patients (21.5%) after the exclusion of 2,768 females who had the outcome prior to the tumor diagnosis, whereas 5,360 male patients (23.6%) were affected and 3,909 males were excluded from the results because they had the outcome prior to the time window. Those excluded subjects were patients, in which the cancer diagnosis was only recorded after the outcome (i.e., death) had occurred at an HCO included in the TriNetX database. The risk difference was statistically significant in a log-rank test (*p* < 0.001). The RR and OR were 0.910 (95% CI [0.879; 0.941]) and 0.885 (95% CI [0.847; 0.924]), respectively. Kaplan-Meier analysis demonstrated a DFS of 71.92% in females, compared to a DFS of 68.29% in males (**Figure 2**), with a statistically significant difference using a log-rank test (*p* < 0.001). The HR was 0.870 (95% CI [0.838, 0.904]).

**Table 2:** Risk difference, risk ratios and odds ratios for disease recurrence in the female and male cohort after propensity score matching.

Cohort statistics				
	Number of patients (N)	Patients with outcome (N)	Risk	
Female	23,810	5,121	0.215	
Male	22,669	5,360	0.236	
Risk analysis				
		95% CI	Z-value	*P*-value
Risk difference	-0.021	-0.029, -0.014	-5.510	0.000
Risk ratio	0.910	0.879, 0.941		
Odds ratio	0.885	0.847, 0.924		

CI, Confidence interval.

The outcome was defined as the occurrence of a secondary malignant neoplasm, secondary lymph node metastasis or a secondary malignant neoplasm of respiratory and digestive organs.

**Figure 3:**
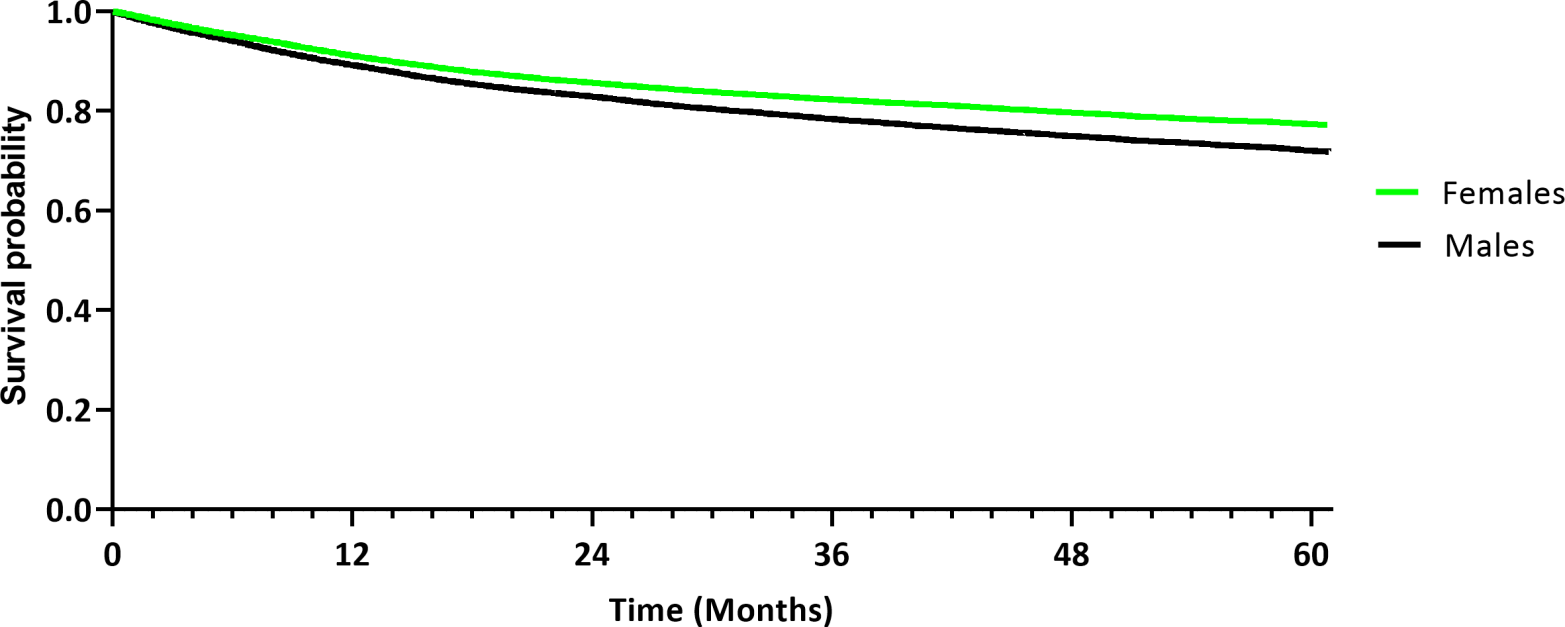
Kaplan-Meier curve demonstrating overall survival of the female and male cohort after propensity score matching.

Next, statistical analysis was performed to assess death and OS in females and males within a five-year period after initial diagnosis (**Table 3**). For further analysis, 85 females and 93 males were excluded from the results because the reported date of death was prior to the documented date of the initial diagnosis. In the female cohort, 3,933 deaths (14.8%) were recorded, whereas 4,700 deaths (17.7%) were recorded in the male cohort. The risk difference was statistically significant in a log-rank test (*p* < 0.001). The RR and OR were 0.837 (95% CI [0.805; 0.870]) and 0.808 (95% CI [0.772; 0.846]), respectively. Kaplan-Meier analysis demonstrated a five-year-OS of 77.08% in the female cohort, compared to a 71.74% OS in the male cohort (**Figure 3**), with a statistically significant difference using a log-rank test (*p* < 0.001). The HR was 0.793 (95% CI [0.760, 0.827]).

**Table 3:** Risk difference, risk ratios and odds ratios for death in the female and male cohort after propensity score matching.

Cohort statistics				
	Number of patients (N)	Patients with outcome (N)	Risk	
Female	26,493	3,933	0.148	
Male	26,485	4,700	0.177	
Risk analysis				
		95% CI	Z-value	*P*-value
Risk difference	-0.029	-0.035, -0.023	-9.038	0.000
Risk ratio	0.837	0.805, 0.870		
Odds ratio	0.808	0.772, 0.846		

CI, Confidence interval.

The outcome was defined as death.

### 3.3 Prognostic role of depression

**Figure 4:**
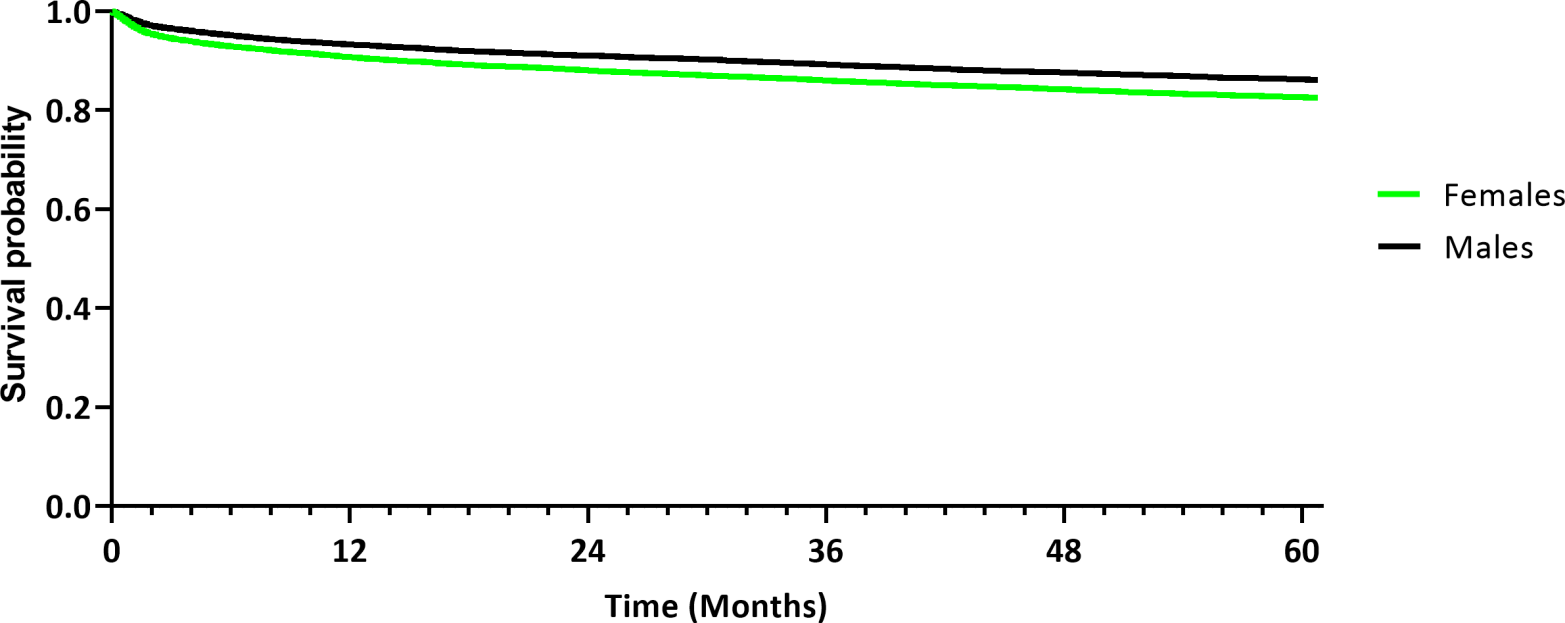
Kaplan-Meier curve demonstrating survival probability of the female and male cohort diagnosed with depression after propensity score matching.

Next, risk of depression and differences in survival were assessed between females and males. The occurrence of depression after the initial diagnosis, as defined per ICD-codes, was assessed between the female and male cohort. After the exclusion of 3,175 female patients and 1,762 male patients because they had the diagnosis of a depression prior to the tumor diagnosis, depression was reported in 2,713 females (11.6%) and 2,111 males (8.5%), with a statistically significant risk difference in a log-rank test (*p* < 0.001). The RR and OR were 1.363 (95% CI [1.291; 1.438]) and 1.410 (95% CI [1.328; 1.497]), respectively (**Table 4**). Kaplan-Meier analysis demonstrated a significantly worse survival probability of 82.48% in females diagnosed with depression (**Figure 4**), compared to an 86.10% survival probability in matched male patients (*p* < 0.001). The HR was 1.341 (95% CI [1.266; 1.419]).

**Table 4:** Risk difference, risk ratios and odds ratios for the diagnosis of depression in the female and male cohort after propensity score matching.

Cohort statistics				
	Number of patients (N)	Patients with outcome (N)	Risk	
Female	23,403	2,713	0.116	
Male	24,816	2,111	0.085	
Risk analysis				
		95% CI	Z-value	*P*-value
Risk difference	0.031	0.025, 0.036	11.287	0.000
Risk ratio	1.363	1.291, 1.438		
Odds ratio	1.410	1.328, 1.497		

CI, Confidence interval.

The outcome was defined as depression after the initial tumor diagnosis.

## 4 Discussion

Better survival and lower cancer mortality in female patients in contrast to male patients have previously been suggested (2), but sex-related aspects and contributing prognostic factors in oral cancer have not been determined. This retrospective analysis evaluated sex disparities in survival in oral cancer patients and examined the potential prognostic role of depression.

This case-control study found a statistically significant difference in survival between females and males diagnosed with oral cancer in a large cohort of 53,156 patients after propensity score matching. In this analysis, females demonstrated significantly greater five-year DFS (71.92%) compared to males (68.29%). OS was also significantly greater in females (77.08%) than in males (71.74%). The results from this study correspond to previously reported differences in age-adjusted cancer mortality related to patients’ sex in other tumor entities, including cancers of the lip, larynx, hypopharynx, esophagus and urinary bladder, with better survival outcomes in females (2). The comparison between the survival rates reported by other studies is difficult due to differences in study design. Survival rates in this retrospective analysis were slightly higher than the previously reported rates of 56% five-year OS and 74% DSS in OSCC patients that were treated with primary surgery (N = 489) (16), which may be due to a smaller sample size, the selected surgical cohort, and differences in patient characteristics.

Sex discrepancies in survival of oral cancer patients remain an issue for discussion. Previous studies identified sex as an independent factor for the prognosis in OPSCC, with controversial reports, however (7, 17). Fakhry et al. reported greater survival in females with HPV-positive OPSCC (17) and laryngeal squamous cell carcinoma (3). Based on a national database, Hunter et al. reported worse survival in females diagnosed with HPV-negative OPSCC, despite lower grade and stage, in a sample size of 2,565 patients (7). OS was found to be 40.7% for females and 54.2% for males at three years. These survival rates were significantly lower than the survival rates found in this study. In tongue squamous carcinoma, male patients had a significantly lower risk of death and recurrence compared to females (8). Mazul et al. reported worse survival in females with HPV-negative OPSCC (18). However, the authors outlined the unreliable HPV status documented in the National Cancer Database as a limitation of their study and risk factors were not considered. Yin et al. reported no sex-related survival difference in HPV-negative OPSCC, which may be attributed to the small sample size (N = 105) (4). Yet, OPSCC and OSCC have different risk factors, tumor biology and survival outcomes, with a better prognosis for HPV-positive head and neck cancers (19).

Previous studies have reported sex-associated effects on treatment outcome in OSCC patients, in which the HPV status is usually negative, but the prognostic value of sex has not been clarified with only limited studies available. Female sex was identified as a significant positive prognostic factor in OSCC in a retrospective single-center study on 147 patients (9). DFS was 51 months in the female cohort, compared to 38 months in the male cohort (*p* < 0.001), but there was no significant difference in DFS and OS between the female and male cohort at five years. The authors attributed these findings to the identified risk factors and the prevalence of advanced lesions in the male group. This single-center study had some limitations, since no matching was conducted, confounding factors were not considered and the sample size was limited. In a small retrospective cohort with 71 women and 142 men with oral tongue cancer, there was no association between patients’ sex and prognosis (20). A retrospective single-center cohort study from Taiwan found no differences in survival between female and male OSCC patients, despite differences in age at diagnosis, anatomical site and risk behavior (21). However, results from this single-center retrospective cohort must be interpreted carefully, given the small patient number in the female cohort (N = 122) and significant differences in the age at diagnosis, anatomic site, and risk behavior. HPV-status of patients included in this trial was not assessed. Small patient numbers limit the level of evidence. To the best of our knowledge, our study is the first to demonstrate better survival outcomes in female oral cancer patients than in male patients in a large, matched patient cohort.

Recent studies have discussed the reasons for sex disparities in cancers. In other tumor entities, diagnosis at earlier stages, comorbidities and better health awareness were proposed as factors for lower incidence and improved survival outcomes in women (22–25). Furthermore, women and men present with different prevailing risk behavior, risk factors and lifestyle choices (10). Known risk factors for the development of oral cancer such as smoking and alcohol consumption are well known, with a higher occurrence in males (1, 26, 27). Moreover, varying genetic predisposition, metabolism and sex hormones may account for sex differences in the development of cancers, including head and neck cancers (10, 28).

This retrospective study suggests a prognostic role of depression after the tumor diagnosis. Survival in females (82.48%) diagnosed with depression after the initial oral cancer diagnosis was worse compared to that in males (86.10%; HR 1.341; *p* < 0.001). An association between the diagnosis of depression and worse prognosis has been reported in other malignancies (13, 29). In patients with B-cell lymphoma, for example, the authors reported worse disease-specific survival outcomes in patients with pre-existing depression and anxiety compared to patients with no mental health disorder (30). Mental health disorders may be associated with delayed treatment, worse treatment adherence and subsequently worse treatment outcomes (14).

The relevance of sex hormones in the development of cancerous lesions in the oral cavity has previously been discussed. In a cohort of 316 patients, the positive expression of estrogen receptor alpha (ERα) was associated with lower OS and RFS, compared to patients with a negative ERα expression status (31). Gender-specific analyses demonstrated a highly significant prognostic effect of the ERα status in male patients, in which positive ERα was associated with worse survival outcomes. In tongue squamous cell carcinoma, the completion of adjuvant radiotherapy was identified as a prognostic factor in females, since females with advanced stage disease who underwent radiotherapy had a better DFS, compared to females who did not undergo adjuvant radiotherapy (8). Similarly, at our institution, female patients who rejected or aborted the recommended adjuvant treatment demonstrated worse survival outcomes than males who did not follow the adjuvant treatment recommendation (Mrosk et al., 2023, manuscript submitted). The diagnosis of a depression may be a contributing factor to poor treatment adherence in cancer patients (32). The relevance of treatment adherence for females diagnosed with oral cancer should be considered in the management of female cancer patients.

An important confounding factor to consider when evaluating results from retrospective cohorts is the previously reported underdiagnosis of depression in male patients as compared to females (33, 34). Furthermore, the retrospective nature of the TriNetX database search does not retrieve clinical details or identify differences in diagnostic methods in the diagnosis of depression, i.e., the type of questionnaires used, among others. The inability to retrieve further clinical data on the diagnosis of depression presents a possible confounding factor of this retrospective study. Future prospective studies should utilize standardized diagnostic techniques in depression. Moreover, if a mental health disorder is suspected or diagnosed, cancer patients should receive individual psycho-oncological consultation (14).

The strengths of this real-world data analysis are the large patient number and the method of propensity score matching to eliminate confounding factors. Matching for age and the well-known risk factors such as smoking and alcohol consumption (26, 27) was conducted. This retrospective study was based on data retrieved from the TriNetX database. HPV-status and other histopathological factors, such as UICC stage, lymph node metastasis, or extracapsular spread (ECS), were not investigated in this study due to the nature of the database. Since the study was based on ICD-10 codes, differences in tumor stage and pathohistological features were not retrieved. Future studies should include the differential analysis of histopathological details, such as tumor stage, grading, HPV status and surgical resection status. As a further limitation, information on the cause of death to clarify whether the cause of death was cancer-related could not be retrieved from the TriNetX database. Moreover, real-world data were retrieved globally and included data provided by HCOs, including countries in Europe, the Middle East, Africa, Asia, as well as North and South America. National and international differences in the management of oral cancer patients and epidemiological discrepancies were not considered in this multi-center analysis.

This retrospective case-matched analysis found improved survival outcomes in females with oral cancer, compared to males. However, the diagnosis of depression was associated with worse survival outcomes in females. We suggest that future prospective and retrospective studies should account for sex disparities in oral cancer patients to elucidate the prognostic role of depression in female cancer patients. Individual psycho-oncological support should be offered in cancer patients, especially if a mental health disorder is suspected or diagnosed. Future studies may consider sex-related aspects in the treatment of oral cancer patients by introducing peri- and postoperative programs for male and female patients, respectively, to improve patient outcomes and quality of life.

## Data availability statement

The datasets presented in this study can be found in online repositories. The datasets used and analyzed can be retrieved from the TriNetX network (https://trinetx.com). Public access to the database is closed. If no access is available, the datasets can be retrieved from the corresponding author based on reasonable request.

## Ethics statement

This study is an analysis of pre-existing, de-identified data. The datasets were retrieved from the TriNetX database. The TriNetX database is compliant with the Health Insurance Portability and Accountability Act (HIPAA) and provides de-identified data as per the standard defined in Section §164.514(a) of the HIPAA Privacy Rule. The process of de-identification is attested to through a formal determination by a qualified expert as stated in Section §164.514(b)(1) of the HIPAA Privacy Rule (https://trinetx.com/real-world-resources/publications/trinetx-publication-guidelines/).

## Author contributions

MH and SK conceived and designed the study. SP was involved in data acquisition. SP and EH were involved in data analysis and interpretation. EH and CD prepared the manuscript and created the figures and tables. All authors (EH, CD, AR, RP, MH, SP, and SK) reviewed the manuscript. All authors contributed to the article and approved the submitted version.

## References

[1] Sung H Ferlay J Siegel RL Laversanne M Soerjomataram I Jemal A. Global cancer statistics 2020: globocan estimates of incidence and mortality worldwide for 36 cancers in 185 countries. CA Cancer J Clin (2021) 71(3):209–49. doi: 10.3322/caac.21660 33538338

[2] Cook MB McGlynn KA Devesa SS Freedman ND Anderson WF. Sex disparities in cancer mortality and survival. Cancer Epidemiol Biomarkers Prev (2011) 20(8):1629–37. doi: 10.1158/1055-9965.EPI-11-0246 PMC3153584 21750167

[3] Li H Li EY Kejner AE. Treatment modality and outcomes in larynx cancer patients: A sex-based evaluation. Head Neck (2019) 41(11):3764–74. doi: 10.1002/hed.25897 31392796

[4] Yin LX D'Souza G Westra WH Wang SJ van Zante A Zhang Y. Prognostic factors for human papillomavirus-positive and negative oropharyngeal carcinomas. Laryngoscope (2018) 128(8):E287–E95. doi: 10.1002/lary.27130 PMC8929688 29536542

[5] D'Souza G Kreimer AR Viscidi R Pawlita M Fakhry C Koch WM. Case-control study of human papillomavirus and oropharyngeal cancer. N Engl J Med (2007) 356(19):1944–56. doi: 10.1056/NEJMoa065497 17494927

[6] Rettig E Kiess AP Fakhry C. The role of sexual behavior in head and neck cancer: implications for prevention and therapy. Expert Rev Anticancer Ther (2015) 15(1):35–49. doi: 10.1586/14737140.2015.957189 25193346 PMC4385715

[7] Hunter WP Harris JA Lee CC Cheng AC Peacock ZS. Females have worse overall and disease-specific survival in human papillomavirus-negative oropharyngeal squamous cell carcinoma. J Oral Maxillofac Surg (2022) 80(7):1260–71. doi: 10.1016/j.joms.2022.03.017 35469827

[8] Tagliabue M D'Ecclesiis O De Berardinis R Gaeta A Martinoli C Piana AF. The prognostic role of sex and hemoglobin levels in patients with oral tongue squamous cell carcinoma. Front Oncol (2022) 12:1018886. doi: 10.3389/fonc.2022.1018886 36457509 PMC9706199

[9] Suresh GM Koppad R Prakash BV Sabitha KS Dhara PS. Prognostic indicators of oral squamous cell carcinoma. Ann Maxillofac Surg (2019) 9(2):364–70. doi: 10.4103/ams.ams_253_18 PMC6933976 31909017

[10] Haupt S Caramia F Klein SL Rubin JB Haupt Y. Sex disparities matter in cancer development and therapy. Nat Rev Cancer (2021) 21(6):393–407. doi: 10.1038/s41568-021-00348-y 33879867 PMC8284191

[11] Linden W Vodermaier A Mackenzie R Greig D. Anxiety and depression after cancer diagnosis: prevalence rates by cancer type, gender, and age. J Affect Disord (2012) 141(2-3):343–51. doi: 10.1016/j.jad.2012.03.025 22727334

[12] Hinz A Herzberg PY Lordick F Weis J Faller H Brähler E. Age and gender differences in anxiety and depression in cancer patients compared with the general population. Eur J Cancer Care (Engl) (2019) 28(5):e13129. doi: 10.1111/ecc.13129 31290218

[13] Pinquart M Duberstein PR. Depression and cancer mortality: A meta-analysis. Psychol Med (2010) 40(11):1797–810. doi: 10.1017/s0033291709992285 PMC2935927 20085667

[14] Davies EA Wang Y-H. Could improving mental health disorders help increase cancer survival? Lancet Haematol (2023) 10(7):e482–4. doi: 10.1016/S2352-3026(23)00156-4 37271157

[15] Schulz KF Altman DG Moher D Group C. Consort 2010 statement: updated guidelines for reporting parallel group randomised trials. J Clin Epidemiol (2010) 63(8):834–40. doi: 10.1016/j.jclinepi.2010.02.005 20346629

[16] Rogers SN Brown JS Woolgar JA Lowe D Magennis P Shaw RJ. Survival following primary surgery for oral cancer. Oral Oncol (2009) 45(3):201–11. doi: 10.1016/j.oraloncology.2008.05.008 18674959

[17] Fakhry C Westra WH Wang SJ van Zante A Zhang Y Rettig E. The prognostic role of sex, race, and human papillomavirus in oropharyngeal and nonoropharyngeal head and neck squamous cell cancer. Cancer (2017) 123(9):1566–75. doi: 10.1002/cncr.30353 PMC5788020 28241096

[18] Mazul AL Colditz GA Zevallos JP. Factors associated with hpv testing in oropharyngeal cancer in the national cancer data base from 2013 to 2015. Oral Oncol (2020) 104:104609. doi: 10.1016/j.oraloncology.2020.104609 32143112

[19] Kobayashi K Hisamatsu K Suzui N Hara A Tomita H Miyazaki T. A review of hpv-related head and neck cancer. J Clin Med (2018) 7(9):241. doi: 10.3390/jcm7090241 30150513 PMC6162868

[20] Garavello W Spreafico R Somigliana E Gaini L Pignataro L Gaini RM. Prognostic influence of gender in patients with oral tongue cancer. Otolaryngol Head Neck Surg (2008) 138(6):768–71. doi: 10.1016/j.otohns.2008.02.026 18503852

[21] Lin NC Hsu JT Tsai KY. Difference between female and male patients with oral squamous cell carcinoma: A single-center retrospective study in Taiwan. Int J Environ Res Public Health (2020) 17(11):3978. doi: 10.3390/ijerph17113978 32512723 PMC7312859

[22] Beisland C Medby PC Beisland HO. Renal cell carcinoma: gender difference in incidental detection and cancer-specific survival. Scand J Urol Nephrol (2002) 36(6):414–8. doi: 10.1080/003655902762467558 12623504

[23] Galdas PM Cheater F Marshall P. Men and health help-seeking behaviour: literature review. J Adv Nurs (2005) 49(6):616–23. doi: 10.1111/j.1365-2648.2004.03331.x 15737222

[24] Dent OF Chapuis PH Renwick AA Bokey EL. The importance of tumor stage and relative survival analysis for the association between sex and survival after resection of colorectal cancer. Ann Surg (2009) 249(3):402–8. doi: 10.1097/SLA.0b013e31819a0469 19247026

[25] Asmis TR Ding K Seymour L Shepherd FA Leighl NB Winton TL. Age and comorbidity as independent prognostic factors in the treatment of non small-cell lung cancer: A review of national cancer institute of Canada clinical trials group trials. J Clin Oncol (2008) 26(1):54–9. doi: 10.1200/JCO.2007.12.8322 18165640

[26] Feller L Chandran R Khammissa RA Meyerov R Lemmer J. Alcohol and oral squamous cell carcinoma. SADJ (2013) 68(4):176–80. 23971298

[27] Jiang X Wu J Wang J Huang R. Tobacco and oral squamous cell carcinoma: A review of carcinogenic pathways. Tob Induc Dis (2019) 17:29. doi: 10.18332/tid/105844 31582940 PMC6752112

[28] Clocchiatti A Cora E Zhang Y Dotto GP. Sexual dimorphism in cancer. Nat Rev Cancer (2016) 16(5):330–9. doi: 10.1038/nrc.2016.30 27079803

[29] Kissane DW. Unrecognised and untreated depression in cancer care. Lancet Psychiatry (2014) 1(5):320–1. doi: 10.1016/S2215-0366(14)70345-1 26360982

[30] Kuczmarski TM Tramontano AC Mozessohn L LaCasce AS Roemer L Abel GA. Mental health disorders and survival among older patients with diffuse large B-cell lymphoma in the USA: A population-based study. Lancet Haematol (2023) 10(7):e530–8. doi: 10.1016/S2352-3026(23)00094-7 37271158 PMC10654921

[31] Doll C Bestendonk C Kreutzer K Neumann K Pohrt A Trzpis I. Prognostic significance of estrogen receptor alpha in oral squamous cell carcinoma. Cancers (Basel) (2021) 13(22):5763. doi: 10.3390/cancers13225763 34830915 PMC8616512

[32] Addington-Hall J. The legacy of cancer on depression and anxiety. Lancet Oncol (2013) 14(8):675–6. doi: 10.1016/S1470-2045(13)70238-9 23759375

[33] Call JB Shafer K. Gendered manifestations of depression and help seeking among men. Am J Men's Health (2018) 12(1):41–51. doi: 10.1177/1557988315623993 26721265 PMC5734537

[34] Faisal-Cury A Ziebold C Rodrigues D Matijasevich A. Depression underdiagnosis: prevalence and associated factors. A Population-Based Study J Psychiatr Res (2022) 151:157–65. doi: 10.1016/j.jpsychires.2022.04.025 35486997

